# Trends of Early Onset Group B Streptococcus infections and Observed Racial and Geographic Disparities Associated with GBS Infections in Tennessee, 2005-2021

**DOI:** 10.1017/ash.2024.297

**Published:** 2024-09-16

**Authors:** Jordan Morris, Erika Kirtz, Daniel Muleta

**Affiliations:** Tennessee Department of Health

## Abstract

**Background:** Group B Streptococcus (GBS) is one of the most common causes of bacterial sepsis in newborns. In 2002, the Center for Disease Control and Prevention (CDC) recommended universal screening of all pregnant women for GBS colonization and administering intrapartum prophylaxis to colonized pregnant women to prevent GBS infection in newborns. To identify racial disparities in GBS infections in Tennessee, we compared the incidence of early-onset GBS infection among Black and White infants from 2005-2021. **Methods:** GBS infections identified from normally sterile sites are reportable in Tennessee. We analyzed GBS data reported to surveillance systems from 2005 to 2021. We linked the surveillance data with the population data to calculate incidence rates. We excluded cases with unknown race status (9%) and other races (0.2%) as we do not have denominator data to calculate the incidence rate. Database linkage and data analyses were performed in SAS V.9.4. **Results:** A total of 399 early-onset GBS cases were reported from 2005–2021; 150 (37.59%) were Black, 212 (53.13%) were White, and 36 (9.02%) were of unknown race, and one (0.20%) reported as Other for race. While the incidence rates of early-onset GBS for all races declined from 0.23 per 1000 live births in 2005 to 0.18 per 1000 live births in 2021, Blacks experienced the largest decline in incidence from 0.6 per 1000 live births in 2005 to 0.37 in 2021. Among Whites, there was a slight decline in 2021 (0.13/1000 live births) compared to the rate in 2005 (0.21/1000 live births). The mean incidence rate of early onset GBS among Blacks (0.52 per 1000 live births) is significantly higher than the mean rates among Whites (0.20 per 1000 live births) (p value < 0 .001) from 2005 to 2021. Shelby County, one of the 95 counties in Tennessee, is predominantly Black (54.6%) and reported 27.8% of all early-onset GBS. **Conclusion:** There was a significant decline in early-onset GBS infections among Blacks and some reductions among Whites, indicating the effectiveness of the prevention strategies. However, Blacks have significantly higher rates than their White counterparts. In addition, 27.8% of the cases are reported from one county, signaling geographic disparities as well. Further investigation is warranted to identify risk factors and causes of observed racial and geographic disparities to help reduce the infection rate among vulnerable populations and high-risk geographic areas.

